# The role of physical activity in the development of first cardiovascular disease event: a tree-structured survival analysis of the Danish ADDITION-PRO cohort

**DOI:** 10.1186/s12933-018-0769-x

**Published:** 2018-09-12

**Authors:** Hanan Amadid, Nanna B. Johansen, Anne-Louise Bjerregaard, Søren Brage, Kristine Færch, Torsten Lauritzen, Daniel R. Witte, Annelli Sandbæk, Marit E. Jørgensen, Dorte Vistisen

**Affiliations:** 1 Clinical Epidemiology, Steno Diabetes Center Copenhagen, Niels Steensens Vej 2-6, 2820 Gentofte, Capital Region Denmark; 2 Department of Public Health, Research Unit & Section of General Practice, Aarhus University, Aarhus, Denmark; 3 Danish Medicines Agency, Copenhagen, Denmark; 4 MRC Epidemiology Unit, University of Cambridge, Cambridge, UK; 5 Danish Diabetes Academy, Odense, Denmark; 6 National Institute of Public Health, University of Southern Denmark, Odense, Denmark

**Keywords:** Cardiovascular disease, Type 2 diabetes, Objective physical activity, Tree-structured survival analysis, Prospective cohort study

## Abstract

**Background:**

Ambiguity exists in relation to the role of physical activity (PA) for cardiovascular disease (CVD) risk reduction. We examined the interplay between PA dimensions and more conventional CVD risk factors to assess which PA dimensions were associated with the first CVD event and whether subgroup differences exist.

**Methods:**

A total of 1449 individuals [median age 65.8 (IQR: 61.2, 70.7) years] with low to high risk of type 2 diabetes and free from CVD from the Danish ADDITION-PRO study were included for survival analysis. PA was measured by individually calibrated heart rate and movement sensing for 7 consecutive days. The associations of different PA dimensions (PA energy expenditure, time spent in light-, moderate- and vigorous intensity PA), sedentary time and other conventional CVD risk factors with the first CVD event were examined by tree-structured survival analysis. Baseline information was linked to data on the first CVD event (ischemic heart disease, ischemic stroke, heart failure, atrial flutter/fibrillation and atherosclerotic disease) and mortality obtained from Danish registers.

**Results:**

During a median follow-up time of 5.5 (IQR: 5.1–6.1) years, a total of 201 individuals (13.9%) developed CVD. Overall CVD incidence rate was 2.6/100 person-years. PA energy expenditure above 43 kJ/kg/day was associated with lower rates of CVD events among participants ≤ 70 years and with HbA_1c_ ≤ 5.7% (39 mmol/mol), systolic blood pressure ≤ 156 mmHg and albumin creatinine ratio ≤ 70 (incidence rates 0.0–0.8/100 person-years).

**Conclusions:**

Any type of PA resulting in increased PA energy expenditure may over time be the best prevention strategy to uphold reduced risk of CVD.

**Electronic supplementary material:**

The online version of this article (10.1186/s12933-018-0769-x) contains supplementary material, which is available to authorized users.

## Introduction

As in the general population, cardiovascular disease (CVD) is a major contributor to morbidity and mortality in individuals with pre-diabetes and type 2 diabetes. However, in these high-risk individuals the risk of developing CVD is greatly increased [1, 2].

Increasing physical activity (PA) levels have shown a substantial favourable impact on type 2 diabetes progression in high-risk individuals [3, 4]. However, the evidence regarding the role of PA in the prevention of CVD in different subgroups is less clear. Observational studies have demonstrated that greater amounts of PA are associated with lower rates of incident and recurrent CVD [5, 6]. However, this is not unambiguously supported by results from large randomized controlled trials of lifestyle interventions in subgroups at high CVD risk [7]. Despite significant improvements of CVD risk factors after increases in PA, these do not seem to convey any appreciable reduction in CVD event rates [7]. The reasons for this inconsistency are not yet fully understood. In contrast, one randomized controlled study found that PA reduced the incidence of CVD among individuals with pre-diabetes after 23 years [8].

Furthermore, the majority of observational studies of the role of PA and the development of CVD have looked only at single measures of PA. Knowledge on how different dimension of PA such as intensity, frequency, duration, and total energy expenditure are associated to CVD risk is therefore limited. In addition, these studies have mainly been conducted in healthy individuals leaving questions unanswered as to whether reported associations are generalizable to high-risk populations. The potential impact of PA on CVD risk may, to an extent, be mediated or modified by other CVD risk factors such as glucose intolerance, obesity, blood pressure, smoking, sex and age but this has rarely been explored. Earlier studies have not examined the potential interplay between different dimensions of PA and CVD risk factors, probably because the conventional statistical methods such as linear regression analyses are primarily focused on mean effects in the entire population and most often do not take into account interactions between multiple explanatory variables [9]. One of the reasons is that assessment of interactions, using the traditional regression models, requires pre-specification of the interaction terms. As the number of explanatory variables in the model increases, the number of possible interactions that can be investigated is large and leads to a complicated model that can be difficult to fit and interpret. Newer tree-based regression techniques provide one approach which account for potential heterogeneity within the population being studied. One of the main advantages of tree-based methods are their data driven exploration of complex interactions among multiple risk factors without any a priori specification. Subgroups are thereby identified by the presence or absence of specific risk factors and who differ in disease risk [10].

Because PA patterns and CVD risk factors are represented in various combinations in individuals, looking into subgroup differences could unveil which role different dimensions of PA in daily living play in cardiovascular health. We recently identified subgroup characteristics and different activity dimensions associated with type 2 diabetes risk [11]. Insight into which combinations of CVD risk/protective factors that are associated to higher or lower CVD incidence could likewise help defining subgroups of the population to which specific interventions, including PA, could be tailored and targeted. We hypothesized that different dimensions of PA may be associated with CVD risk in different ways. Moreover, we speculated whether the association between PA dimensions and CVD are the same in every individual or whether subgroup differences exist. Thus, we applied a tree-based approach with an extension to survival data, *tree*-*structured survival analysis* (TSSA) [12] with the purpose to examine the interplay between objectively measured PA dimensions and more conventional CVD risk factors in a population with low to high risk for type 2 diabetes [13], to assess which PA dimensions are associated with risk of first CVD event and whether subgroup differences exist.

## Methods

### Study design and participants

Participants for this prospective observational study were recruited between 2009 and 2011 from the Danish arm of the population-based ADDITION-Europe study (ADDITION-DK), a stepwise screening program for type 2 diabetes in primary care [14]. ADDITION-PRO was the follow-up health examination of individuals without type 2 diabetes at screening but with low to high risk of type 2 diabetes defined by the screening procedure in ADDITION-DK. In brief, the stratification of type 2 diabetes risk in ADDITION-DK was based on a Danish diabetes risk score questionnaire [15]. Participants were asked to indicate known risk factors for type 2 diabetes including age, sex, BMI, known hypertension, family history of type 2 diabetes, gestational diabetes and leisure time PA. Participants with a risk score ≥ 5 points (maximum 15 points) were invited to continue in the stepwise screening programme, which included random blood glucose and glycated haemoglobin A_1c_ (HbA_1c_) testing, a fasting blood glucose test, and an oral glucose tolerance test. World Health Organisation criteria were used to diagnose type 2 diabetes [16]. The sampling frame for the ADDITION-PRO study consisted of participants classified in groups of increasing type 2 diabetes risk according to the type 2 diabetes risk score and glycaemic status: low type 2 diabetes risk (< 5 points on type 2 diabetes risk score); high type 2 diabetes risk (≥ 5 points on type 2 diabetes risk score) with (i) normoglycemia; (ii) isolated impaired fasting glucose; (ii) isolated impaired glucose tolerance; and (iv) combined impaired fasting glucose and impaired glucose tolerance. In total, 2082 individuals agreed to participate in the ADDITION-PRO health examination which serves as baseline for the current study. The ADDITION-PRO study focuses on the aetiology, pathophysiology, complications and comorbidities of type 2 diabetes and is characterized by an extensive phenotyping. The study has been described previously [13].

### Measurements and definitions

#### Cardiometabolic risk factors

In the ADDITION-PRO cohort, cardiometabolic risk factors were assessed at baseline in 2009–2011 and included biochemical, anthropometric, and behavioural assessments as part of the larger health examination procedure. The detailed methods of measurements or calculations of HbA_1c_, total cholesterol, HDL-cholesterol, LDL-cholesterol, triglycerides, plasma creatinine, urinary albumin creatinine ratio and estimated glomerular filtration rate as well as body mass index (BMI) and diastolic and systolic blood pressure have been previously published [13].

#### Other covariates

Information on age and sex were obtained from the unique Danish civil registration number. Information on smoking, occupation and medication use was collected from self-administered questionnaires at baseline. Smoking was categorized as current smokers and non-smokers (including prior smokers). Occupation was categorized into those who were employed and those who were unemployed (including housewives, retired individuals and individuals receiving government provisions). Medication was categorized into CVD protective medications: use of any kind of anti-diabetic drug, anti-hypertensive drug, lipid-lowering drug, and/or aspirin.

#### Physical activity

Physical activity was measured using a combined accelerometer and heart rate monitor (ActiHeart, CamNTech, Cambridge, UK) [17]. The monitor measures uniaxial acceleration and heart rate independently. The monitor was fitted horizontally on the participant’s chest with two standard electrocardiogram electrodes (Maxensor, Alton, UK), one at the lower part of the sternum and the other one on the same horizontal level, on the left side, as laterally as possible. Each participant wore a monitor continuously for seven consecutive days. An 8-min submaximal step test was performed to individually calibrate heart rate to PA intensity [18]. For participants who did not complete an individual calibration due to physical impairment or certain cardiovascular conditions, e.g. angina pectoris, valid calibration tests in the rest of the sample (*n* = 1046) were used to derive a group calibration equation adjusted for age, sex, beta-blocker use, and sleeping heart rate, for the translation of heart rate into activity intensity. Heart rate data collected during the free-living period was processed using noise classification followed by Gaussian robust regression [19], and average activity intensity was estimated using a branched equation framework [20].

The energy cost of any daily PA beyond sleep expressed as PA energy expenditure (PAEE) (kJ/kg/day), in addition to sedentary time excluding self-reported sleep, and time spent in light-, moderate- and vigorous PA were derived, whilst minimizing diurnal information bias [21]. Time spent at various metabolic equivalent of task (MET) levels represent the PA intensity distribution across a 24 h spectrum [22]. Sedentary time was defined as an intensity of < 1.5 METs, light PA as 1.5–3.0 METs, moderate-to-vigorous PA (MVPA) as ≥ 3.0 METs and vigorous PA as an intensity ≥ 6.0 METs, using a fixed value of 20.35 J/ml O_2_ × 3.5 ml O_2_/min/kg to define 1 MET. To indicate the degree of activity accumulation occurring in bouts, time spent in continuous MVPA lasting ≥ 10 min was derived as well as two categorical variables of meeting Danish MVPA-guidelines [23]: 30 min of MVPA per day regardless of bout duration and 30 min of MVPA per day in bouts of at least 10 min. To ensure the best PA estimates, only measures from participants with a minimum of 48 h of ActiHeart wear time were considered valid for the present analysis.

#### Ascertainment of cardiovascular disease events

All Danish residents have a unique civil registration number recorded in the Danish Civil Registration System [24]. The registers in Denmark are nationwide and cover all residents. Clinical and PA data were linked to data on diagnoses and procedures from the Danish National Patient Register [25] using this civil registration number. Similarly, data on death and emigration was obtained from the Central Person Register [26]. We defined CVD as a composite outcome of events of ischemic heart disease, ischemic stroke, heart failure, atrial fibrillation/flutter and atherosclerotic disease. See Additional file 1: Table S1 for the specific *International Classification of Diseases* codes and Danish procedure codes used.

### Statistical analyses

All the participants were followed from baseline of the study (date of the ADDITION-PRO clinical health examination) until first event of CVD, emigration, death or the end of the follow-up period (date of register extraction; May 4, 2016), whichever came first. Participants with a previous CVD diagnosis in the registers at baseline were excluded (n = 400). Also, participants with less than 48 h of monitor wear or in whom PA measurement failed were excluded (n = 233), leaving a total of 1449 individuals with 7720 person-years to be included in the present analysis.

Descriptive characteristics of the study sample at baseline by CVD status at end of follow-up were summarized as medians with inter quartile ranges (IQR) for non-normal data, means with standard deviations (SD) for normally distributed data or as numbers and percentages. To examine differences in characteristics between the groups, we conducted χ^2^ tests for categorical variables and t-tests for normally distributed data. In non-normal data, differences were tested using the Man-Whitney U test.

The interplay between PA and conventional CVD risk factors as well as the association between PA dimensions and CVD incidence was assessed by tree-structured survival analysis (TSSA) (“party” package in R) [12, 27]. TSSA extends tree-based methods to survival data taking into account time to event. Briefly, a survival tree is constructed using recursive partitioning [28]. The recursive partitioning algorithm identifies the risk factor and the split in this factor which gives the maximal difference in CVD event rates between the two resulting subgroups. This procedure is applied recursively until the tree has been grown to an optimal number of “terminal leaves”. The terminal leaves comprise subgroups in the study population, characterized by a different sequence of classifications by the risk factors included in the model.

In the TSSA, the following dimensions of PA were considered as risk factors for CVD: (a) overall PAEE (b) light PA (c) MVPA (d) MVPA accumulated in bouts ≥ 10 min and (e) vigorous PA (f) meeting Danish MVPA-guidelines of 30 min of MVPA per day (bouted and non-bouted). Also, sedentary time was considered a risk factor for CVD. Additional conventional CVD risk factors included for analysis were: sex, age categorized in 5-year age bands (45–49, 50–54, …, 75–79, years) and smoking, in addition to BMI, systolic and diastolic blood pressure, HbA_1c_, total-, HDL- and LDL-cholesterol and triglycerides as continuous variables. Beyond their role as markers for kidney disease risk, albumin creatinine ratio and estimated glomerular filtration rate are well-established powerful independent risk factors for cardiovascular morbidity and mortality across populations with or at risk of type 2 diabetes. We included these renal biomarkers as continuous variables in the model.

Because a large proportion of the study population was using CVD protective medication at baseline, we did a sensitivity analysis, deriving a TSSA including medication use as a potential risk factor for incident CVD.

Incidence rates (IR) per 100 person-years with 95% confidence intervals (CI) for the composite CVD outcome were calculated for the entire population and for the final subgroups that emerged from the TSSA by Poisson regression analyses using log-person time as the offset variable.

Data management was performed in SAS version 9.4. Statistical analyses were performed in R version 3.1.3 (The R Foundation for Statistical Computing, http://www.R-project.org).

## Results

### Characteristics of the study population

Population age ranged from 45 to 79 years with men constituting 51% of the population. Median follow-up was 5.5 years (IQR: 5.1–6.1), during which 201 (13.9%) individuals developed CVD, 57 died and 3 emigrated. The overall incidence rate for CVD was 2.6 per 100 person-years. Ischemic heart disease totalled 43% of the CVD events, ischemic stroke was 20%, atrial fibrillation/flutter 30%, heart failure was 1%, and atherosclerotic disease was 6% of the CVD events.

Participants who developed CVD during follow-up were older, had a larger proportion of men and a higher proportion of participants in anti-hypertensive and/or aspirin treatment compared to participants who did not develop CVD (Table 1). There were no baseline differences in the proportions of smokers or participants with normal glucose tolerance, pre-diabetes or type 2 diabetes between participants who did and did not develop CVD. The metabolic profile among participants who developed CVD was less favourable with higher measures of BMI, systolic blood pressure and HbA_1c_. Among participants who did not develop CVD, total PAEE was higher and more time was spent in both light PA, MVPA and MVPA bouts of more than 10 min compared to participants who developed CVD. None of the participants accumulated any time in vigorous PA. Participants who developed CVD spent more time sedentary (≈ 30 min) than participants who did not develop CVD.

**Table 1:** Baseline characteristics of the study population by CVD event occurrence

	No CVD event	CVD event
N	1248	201
Male sex (%)	622 (49.8)	119 (59.2)*
Age (years)	65.4 [60.7, 70.2]	68.6 [63.3, 72.1]**
Employed (% yes)^a^	529 (42.4)	72 (35.8)
Smoking (% yes)	191 (15.3)	36 (17.9)
Clinical measures		
Body mass index (kg/m^2^)	27.0 [24.1, 30.4]	27.6 [25.3, 30.5]*
Systolic blood pressure (mmHg)	133 (17.3)	136 (17.8)*
Diastolic blood pressure (mmHg)	82 (10.4)	83 (10.5)
Total cholesterol (mmol/l)	5.5 (0.9)	5.5 (1.0)
High density lipoprotein cholesterol (mmol/l)	1.5 [1.1, 1.9]	1.5 [1.2, 1.6]*
Low density lipoprotein cholesterol (mmol/l)	3.3 (0.8)	3.2 (1.1)
Triacylglycerol (mmol/l)	1.0 [0.7, 1.5]	1.2 [0.9, 1.6]
HbA1c (%)	5.7 [5.5, 6.0]	5.8 [5.5, 6.1]**
HbA1c (mmol/mol)	39 [37, 42]	40 [37, 43]**
Albumin creatinine ratio	87 [49, 114]	86 [35, 114]
Estimated glomerular filtration rate (ml/min/1.73m^2^)	79.4 [70.6, 88.0]	77.0 [68.0, 87.0]*
Medication use		
Any anti-diabetic drug (%)	61 (4.9)	12 (6.0)
Any anti-hypertensive drug (%)	474 (38.3)	100 (50.0)**
Any lipid-lowering drug (%)	303 (24.5)	55 (27.5)
Aspirin (%)	164 (13.2)	45 (22.5)**
Physical activity behavior		
Monitor wear time (days)	6.6 (1.3)	6.7 (1.2)*
Physical activity energy expenditure (kJ/kg/day)	31.0 [22.0, 42.0]	28.0 [21.0, 39.0]*
Light intensity physical activity (h/day)	4.8 (1.8)	4.5 (1.7)
MVPA (min/day)	29.8 [12.0, 56.3]	24.0 [7.3, 44.7]**
MVPA in bouts ≥ 10 min (min/day)	8.2 [0.0, 24.4]	4.4 [0.0, 18.7]*
MVPA guidelines ≥ 30 min/day (%)	532 (42.6)	72 (35.8)
Vigorous physical activity (min/day)	0.0 [0.0, 0.3]	0.0 [0.0, 0.1]
Awake sedentary time (h/day)	12.0 (2.2)	12.4 (2.3)*

Data are median [IQR], mean (SD) or n (%)

*HbA1c* glycated hemoglobin A1c, *MVPA* moderate-to-vigorous physical activity

* For p-value < 0.05 and ** for < 0.01

^a^Employed includes the self-employed. Out of work includes the unemployed, housewives, retired individuals and individuals receiving government provisions

### Tree-structured survival analysis

**Fig. 1:**
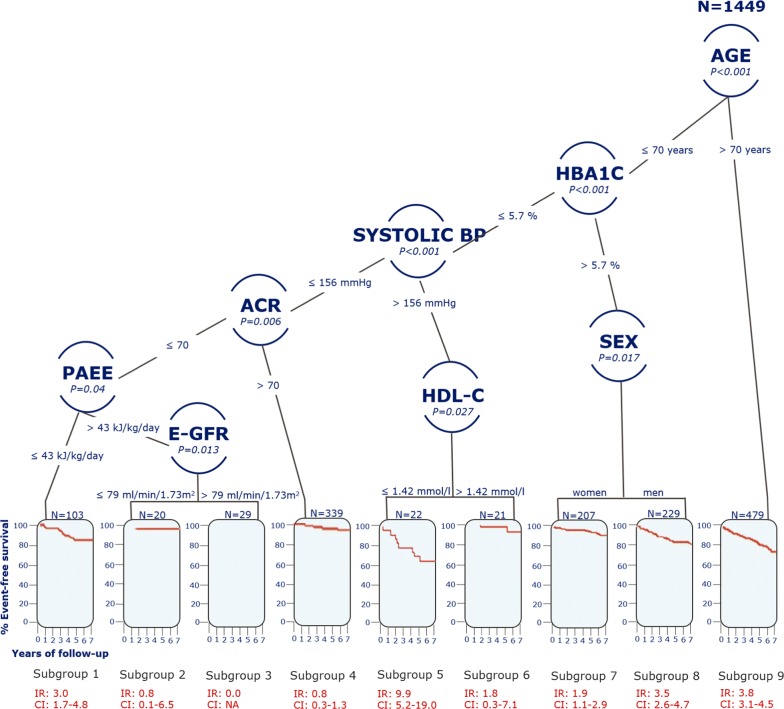
Tree-structured survival analysis for first cardiovascular disease event in the ADDITION PRO cohort study. *AGE* age at baseline, *HBA1c* glycated hemoglobin A1c, *SYSTOLIC BP* systolic blood pressure, *SEX* sex, *HDL-C* HDL-cholesterol, *ACR* albumin creatinine ratio, *PAEE* physical activity energy expenditure, *E-GFR* estimated glomerular filtration rate, *IR* incidence rate per 100 person-years, *CI* 95% confidence interval

The TSSA retained eight factors associated with incident CVD: age, HbA_1c_, systolic blood pressure, sex, albumin creatinine ratio, HDL-cholesterol, PAEE and estimated glomerular filtration rate (Fig. 1). Age, HbA_1c_ and systolic blood pressure were the strongest risk factors associated with incident CVD as these were ranked highest in the tree. Interactions between the retained risk factors associated with CVD incidence and splits in these defined nine subgroups. To interpret the tree, risk factor splits and branches are followed through the tree from the top “root node” which contains all the participants until a “terminal leaf” constituting a subgroup is reached. For example, risk factors that are associated with CVD and define subgroup 1 are age (≤ 70 years), HbA_1c_ [≤ 5.7% (39 mmol/mol)], systolic blood pressure (≤ 156 mmHg), albumin creatinine ratio (≤ 70) and PAEE (≤ 43 kJ/kg/day) while for subgroup 7 and 8 age (≤ 70 years), HbA_1c_ [> 5.7% (39 mmol/mol)], and sex was associated with CVD, with men having the highest incidence rates for CVD.

Among the analysed PA dimensions, only PAEE was associated with incident CVD and solely among participants aged ≤ 70 years, and having HbA_1c_ ≤ 5.7% (39 mmol/mol), systolic blood pressure ≤ 156 mmHg and albumin creatinine ratio ≤ 70. The optimal split in PAEE was at 43 kJ/kg/day resulting in two branches. One branch contained participants with PAEE levels ≤ 43 kJ/kg/day [subgroup 1; IR 3.0 (CI 1.7–4.8)] and the second included participants with PAEE > 43 kJ/kg/day. In the high PAEE subgroup, estimated glomerular filtration rate was also associated with CVD development and thereby formed two further subgroups [subgroups 2 and 3; IR 0.8 (CI 0.1–6.5) and 0.0 (CI: NA)].

Descriptive characteristics of all the CVD risk groups are displayed in Tables 2 and 3.

**Table 2:** Subgroup CVD risk factor characteristics

Subgroups	1	2	3	4	5	6	7	8	9
N	103	20	29	339	22	21	207	229	479
Events (%)	16 (15.5)	1 (5.0)	0 (0.0)	14 (4.1)	9 (40.9)	2 (9.5)	22 (10.6)	43 (18.8)	94 (19.6)
Male sex (%)	45 (43.7)	8 (40.0)	16 (55.2)	170 (50.1)	16 (72.7)	10 (47.6)	0 (0.0)	229 (100.0)	247 (51.6)
Age (years)	64.4 [59.9, 66.3]	60.0 [53.5, 62.7]	62.2 [57.3, 66.0]	62.0 [57.8, 65.3]	66.0 [63.6, 68.3]	63.3 [62.1, 66.2]	63.1 [58.5, 65.7]	63.3 [59.6, 66.0]	72.5 [70.6, 75.0]
Smoking (% yes)	13 (12.6)	2 (10.0)	6 (20.7)	34 (10.0)	4 (18.2)	1 (4.8)	44 (21.3)	61 (26.6)	62 (12.9)
Employed (% yes)	56 (54.4)	15 (75.0)	15 (51.7)	200 (59.0)	9 (40.9)	11 (52.4)	86 (41.5)	141 (61.6)	68 (14.2)
Body mass index (kg/m^2^)	26.1 [23.3, 28.9]	23.3 [22.0, 25.8]	24.5 [22.6, 27.3]	26.2 [23.6, 29.3]	29.2 [26.0, 30.7]	27.8 [25.0, 31.0]	29.0 [25.4, 33,0]	29.1 [26.7, 31.7]	26.5 [24.2, 29.5]
Systolic blood pressure (mmHg)	129 (14)	127 (15)	130 (14)	127 (14)	167 (8)	167 (12)	131 (17)	137 (16.3)	135 (17.0)
Diastolic blood pressure (mmHg)	81 (9)	79 (11)	82 (9)	81 (9)	100 (6)	95 (11)	83.2 (9.8)	84.6 (10.1)	80.1 (10.3)
Total cholesterol (mmol/l)	5.7 (0.8)	5.4 (0.7)	5.8 (1.0)	5.4 (1.0)	5.8 (0.9)	6.0 (1.0)	5.3 (1.0)	5.1 (1.0)	5.3 (1.1)
High density lipoprotein cholesterol (mmol/l)	1.5 [1.2, 2.0]	1.5 [1.4, 2.2]	1.7 [1.4, 2.0]	1.6 [1.3, 1.8]	1.3 [1.0, 1.2]	1.5 [1.4, 2.2]	1.5 [1.1, 1.8]	1.1 [1.0, 1.5]	1.6 [1.2, 1.8]
Low density lipoprotein cholesterol (mmol/l)	3.3 (0.7)	3.1 (0.5)	3.5 (0.9)	3.4 (0.9)	3.7 (0.7)	3.6 (0.6)	3.2 (1.0)	3.1 (1.1)	3.3 (1.0)
Triacylglycerol (mmol/l)	1.0 [0.9, 1.3]	0.7 [0.5, 1.3]	1.1 [0.6, 1.5]	1.0 [0.8, 1.3]	1.5 [1.1, 2.3]	0.8 [0.7, 1.0]	1.2 [1.0, 1.8]	1.4 [1.1, 2.1]	1.1 [0.7, 1.5]
HbA1c (%)	5.3 [5.3, 5.5]	5.4 [5.4, 5.5]	5.4 [5.3, 5.6]	5.4 [5.2, 5.5]	5.6 [5.3, 5.6]	5.4 [5.4, 5.5]	6.0 [5.7, 6.1]	6.0 [5.8, 6.3]	5.8 [5.4, 6.0]
Albumin creatinine ratio	29 (20)	38.3 (16.7)	27.5 (19.6)	105.6 (21.0)	76.0 (44.0)	74.2 (43.2)	77.2 (46.4)	84.3 (40.1)	79.2 (43.3)
Estimated glomerular filtration rate (ml/min/1.73m^2^)	80.0 [72.0, 89.0]	74.0 [70.8, 75.4]	88.0 [84.0, 93.0]	81.0 [72.0, 89.0]	77.4 [69.3, 88.0]	78.0 [73.0, 89.0]	82.0 [71.0, 91.4]	86.0 [76.0, 92.0]	74.0 [66.0, 84.0]
Any anti-diabetic drug (%)	0 (0.0)	0 (0.0)	0 (0.0)	6 (1.8)	1 (4.5)	0 (0.0)	22 (10.8)	26 (11.4)	18 (3.8)
Any anti-hypertensive drug (%)	38 (36.9)	5 (25.0)	4 (13.8)	98 (29.3)	9 (40.9)	10 (47.6)	98 (48.0)	96 (41.9)	216 (45.5)
Any lipid-lowering drug (%)	15 (14.6)	2 (10.0)	2 (6.9)	39 (11.6)	4 (18.2)	1 (4.8)	80 (39.2)	81 (35.4)	134 (28.2)
Aspirin (%)	9 (8.7)	0 (0.0)	1 (3.4)	26 (7.8)	4 (18.2)	5 (23.8)	36 (17.6)	43 (18.8)	85 (17.9)

Data are median [IQR], mean (SD) or n (%)

*HbA1c* glycated hemoglobin A1c

* For p-value < 0.05 and ** for < 0.01

^a^Employed includes the self-employed. Out of work includes the unemployed, housewives, retired individuals and individuals receiving government provisions

**Table 3:** Subgroup physical activity characteristics

Subgroups	1	2	3	4	5	6	7	8	9
Monitor wear time (days)	6.6 (1.4)	6.2 (1.8)	6.6 (1.4)	6.4 (1.2)	6.5 (1.6)	6.7 (1.0)	6.50 (1.2)	6.76 (1.20)	6.71 (1.24)
PAEE (kJ/kg/day)	28.0 [23.0, 36.0]	50.4 [46.0, 55.3]	52.0 [49.0, 58.0]	35.0 [25.0, 46.0]	31.0 [21.0, 38.4]	28.0 [22.0, 41.0]	28.0 [20.0, 36.0]	35.0 [26.0, 44.0]	26.0 [20.0, 36.0]
Light intensity physical activity (h/day)	4.3 (1.4)	7.0 (1.6)	7.3 (1.4)	5.0 (1.9)	4.6 (1.6)	4.1 (1.9)	4.7 (1.8)	5.32 (1.85)	4.42 (1.75)
MVPA (min/day)	30.7 [15.9, 43.5]	79.3 [64.6, 93.6]	84.5 [70.8, 104.3]	38.2 [17.9, 69.0]	20.0 [6.0, 52.7]	23.4 [8.7, 39.3]	21.3 [8.3, 45.0]	33.9 [14.4, 60.5]	18.6 [8.4, 39.2]
MVPA in bouts ≥ 10 min (min/day)	6.7 [0.0, 17.5]	28.6 [16.5, 52.4]	32.1 [21.8, 57.1]	14.2 [3.3, 32.2]	8.5 [0.0, 23.7]	3.3 [0.0, 24.1]	5.3 [0.0, 16.7]	8.53 [1.3, 25.9]	5.11 [0.0, 15.9]
MVPA guidelines ≥ 30 min/day (%)	44 (42.7)	16 (80.0)	27 (93.1)	181 (53.4)	10 (45.5)	6 (28.6)	70 (33.8)	111 (48.5)	139 (29.0)
Vigorous physical activity (min/day)	0.0 [0.0, 0.7]	2.2 [0.0, 13.7]	0.1 [0.0, 1.7]	0.0 [0.0, 2.7]	0.0 [0.0, 0.2]	0.0 [0.0, 0.2]	0.0 [0.0, 0.0]	0.00 [0.00, 0.42]	0.00 [0.00, 0.00]
Awake sedentary time (h/day)	12.4 (1.7)	10.5 (3.1)	9.0 (2.0)	11.7 (2.2)	12.0 (1.8)	12.5 (2.0)	12.5 (2.3)	11.8 (2.4)	12.7 (2.0)

*PAEE* physical activity energy expenditure, *MVPA* moderate-to-vigorous physical activity

* For p-value < 0.05 and ** for < 0.01

After adding CVD protective medications use as risk factors for CVD in the subsidiary TSSA (sensitivity analysis), no dimension of PA was associated with CVD. Risk factors most strongly associated with incident CVD were age, use of aspirin, HbA_1c_, use of any anti-hypertensive drugs and HDL-cholesterol (Additional file 2: Figure S1).

## Discussion

We followed 1449 Danish men and women free of CVD at baseline for a median time of 5.5 years regarding first CVD events. We investigated the role of various dimensions of PA and the interplay with other CVD risk factors associated with first CVD event and identified the dimension of PA with potential benefit to cardiovascular health. The TSSA identified nine subgroups through varying combinations of risk factors and they corresponded to three levels of CVD risk. The lowest risk groups (subgroup 2, 3 and 4; IR 0.0–0.8) were characterized by younger age, higher PA level and a relatively good CVD risk factor profile (Tables 2 and 3). Five moderate CVD risk groups (subgroup 1, 6, 7, 8; IR 1.8–3.8) had varying PA characteristic and varying levels of different CVD risk factors. Finally, one high risk group (subgroup 5; IR 9.9) was characterized primarily by levels of systolic and diastolic blood pressure, LDL-cholesterol and triacylglycerol which were the highest of all of the nine groups identified by the TSSA.

Interestingly, we found PAEE to be the sole activity dimension with an association to incident CVD, independent of other CVD risk factors, and only among individuals with a relatively healthy CVD profile; this subgroup was comprised of individuals aged ≤ 70 years with HbA_1c_ levels ≤ 5.7% (39 mmol/mol), systolic blood pressure ≤ 156 mmHg and albumin creatinine ratio ≤ 70 (Fig. 1).

### Physical activity, CVD risk factors and modulation of CVD risk

Cardiovascular disease is a multifactorial disease involving many risk factors. PA positively affects the same risk factors that contribute to cardiovascular risk such as BMI, blood pressure, lipids, insulin sensitivity, blood coagulability and cardiac function (increased myocardial oxygen supply, improved myocardial contraction, and electrical stability) [29]. Detailed molecular mechanisms of the PA-mediated prevention of CVD are not fully uncovered. However, recently it has been shown that modulations of microRNAs in an immediate response to PA are able to induce significant cardio-protection [30]. Adherence to a physically active lifestyle over the long term may therefore modify risk factors for CVD and subsequently prime individuals for a more favourable CVD profile which in turn could lead to a lower cardiovascular risk.

### Differential impact of physical activity on CVD risk in subgroups

We considered possible explanations for the lack of significant associations between any of the PA dimensions and incident CVD in the other subgroups with relatively unfavourable CVD profiles. Our diabetes risk-stratified study population makes a direct comparison with other study populations challenging. Nevertheless, our results corroborate those studies examining the impact of PA on CVD risk. Previous evidence does support more favourable effects of PA on CVD risk among low risk compared to high risk subgroups [31]. In the Look AHEAD Study there was no significant effect of a lifestyle intervention including PA on CVD events [32]. However, using a recursive partitioning approach as we did, Baum and colleagues recently examined whether the intervention effect differed between different subgroups of study participants. Findings showed that individuals with low HbA_1c_ and a good general health experienced the most significant reduction in CVD events from the intervention [33]. These findings support the notion that the impact of PA in subgroups with unfavourable CVD profiles is attenuated and thus may emphasize the importance of being physically active when CVD risk is still low. Another reason why we found no PA dimension associated with CVD in the subgroups with unfavorable CVD profiles, could be that individuals in these subgroups show homogeneous PA patterns. Lack of variation in PA patterns (and therefore lack of an independent explanatory effect) would not allow for any of the PA dimensions to further discriminate CVD event risk between mutually exclusive subgroups. Therefore, a potential CVD benefit from any of the PA dimensions cannot be ruled out in these subgroups.

There is little evidence about the potential interaction of medication use in relation to PA and CVD. While some studies find no differential effect of PA between user and non-user of CVD protective medication [34], others report of non-significant associations between PA and CVD after adjustment for CVD drug use [35]. However, PA is known to be highly and inversely correlated to the use of CVD protective medication [36]. In our study, the subgroups with the highest incidence of CVD were comprised of individuals who on average were more sedentary and with higher proportions of individuals who received various CVD protective medications compared to the other subgroups. As a result CVD protective medication use may be a proxy for physical inactivity, which could explain why inclusion of medication use in the statistical model resulted in no association of PA with CVD incidence.

### Implications of increasing physical activity energy expenditure and for future physical activity guidelines

Overall PA levels in the study population as a whole were low to very low. In accordance with studies in populations similar to ours, we also found that more than 70% of the time awake was spent sedentary and only 3% of the time was spent on MVPA [37]. Although the study population accumulated a median of 28 min/day of MVPA, the large fraction of time spent sedentary resulted in an overall low total PAEE (median 30 kJ/kg/day). As such high levels of sedentary behaviour may coexist with meeting recommended levels of MVPA. However, accumulating evidence suggests that sedentary behaviour, independent of PA levels, may be associated with an increased risk of cardiovascular disease and a variety of other health problems [38]. Based on the evidence for the benefit of regular PA in primary and secondary prevention of CVD, the Danish and international PA guidelines for older adults (age ≥ 65) (and for adults aged 50–64 with clinically significant chronic conditions) emphasize participation in minimum 30 min/day of MVPA in bouts of more than 10 min [23, 39–41]. However, individuals with or at risk of type 2 diabetes generally have low levels of MVPA and spend a large portion of their day being sedentary [42]. Due to various barriers known to limit and interfere with the ability or motivation to adhere to PA recommendations, high risk individuals do not achieve increases in MVPA of sufficient magnitude to confer health benefit [43, 44]. For example, individuals with uncomplicated type 2 diabetes exhibit an attenuated increase in stroke volume during PA attributed to impaired left ventricular filling at higher heart rates [45]. However, the view that PA has to be of moderate-to-vigorous intensity in order to yield cardiovascular risk reduction has been debated [46–48]. Our findings suggest that increases in overall PAEE may have health benefits regardless of how that increase is achieved. Although higher intensity PA may offer superior cardiovascular benefits in terms of improved cardiorespiratory fitness [49], it may not be a prerequisite for CVD risk reduction. In fact, regular PA of even low-to-moderate intensity, typical of everyday life has shown favourable effects on cardio metabolic risk factors including HbA_1C_ as well as CVD events [6, 49, 50]. We found that PAEE above 43 kJ/kg/day was associated with lower rates of CVD events. In a comparable study population using the same PA monitor to validate a four-category PA index, PAEE above 43 kJ/kg/day categorized individuals as being moderately active [51]. This activity level typically represents a lifestyle for an individual with sedentary work-conditions but who are engaged in leisure time PA of > 3.5 and ≤ 7.0 h/week [51]. As such, our finding of an association between PAEE and CVD incidence has important implications for public health. In most populations, PAEE is mainly composed of “daily activities” across the energy expenditure spectrum, rather than regular MVPA sessions. Targeting increases in PAEE can be achieved by seeking out opportunities to be more physically active in all types of activities in daily living (e.g. walking, household tasks, stair climbing). Because these activities need not necessarily be of high intensity or accumulated in a structured manner, it may be a more palatable message and feasible strategy to managing CVD risk especially for those who perhaps struggle to increase their PA level through MVPA. This argument of increasing PAEE and not just MVPA is reflected in the inclusion of sedentary behaviour recommendations and the often included “more is better” message in several recently updated PA guidelines [52].

### Strengths and limitations

Using the ActiHeart monitor for objective PA measurement in the ADDITION-PRO study overcame the limitations, bias and poor validity associated with self-report [17, 53–55]. In addition, the individually calibrated and combined measures from heart rate and accelerometry has shown more accurate measures of PAEE in comparison to measures obtained by solely heart rate or solely accelerometry [18, 20, 56]. The validity and reliability of the ActiHeart monitor has been successfully tested within various populations in several studies [17, 57, 58]. The ActiHeart monitor is limited by its relatively lower feasibility and costs when used in large-scale epidemiological studies. The individual calibrations of the monitor, data cleaning and processing are time consuming and require trained staff to obtain good quality measures. Furthermore, our measure of free-living PA takes into account all types and intensities of activities performed in daily life and not only structured exercise and related structured activities. This is important in a study population that spends a considerable amount of time in low intensity, unplanned, and unstructured activities that are difficult to quantify by self-report. However, the interpretation of our results is somewhat limited by the single baseline measurement of PA. Repeated PA measures would allow for examination of whether changes in PA per se are associated with CVD. In addition, PA measures may be overestimated among participants who did not perform a step-test but for whom a group calibration value was assigned, since these participants were less healthy participants compared to the rest of the sample.

The complex interplay between PA and CVD risk factors in the development of CVD argue for studying the association of PA and several CVD risk factors with incident CVD together rather than adjusting for these factors to study the other. We applied a novel approach using TSSA to assess the possible heterogeneity of the role of PA dimensions affecting CVD development. TSSA has highly comparable metrics compared to conventional regression and conveys results in a visual and an easily interpretable manner [59]. One constraint for the present study is collinearity between the modelled PA dimensions. However, collinearity is a bigger concern with the use of conventional regression models where correlation makes the estimated regression coefficients more difficult to interpret and increases their variability. In the TSSA, at each step, the most predictive or informative explanatory variable is selected to split a node of the tree. After splitting on the variable, its explanatory power is depleted, and other variables may be selected in subsequent steps. If two or more highly correlated explanatory variables are almost equally informative, however, the most statistically significant one is selected. More important, given the data-driven nature of the TSSA, external validation of the results is essential. Furthermore, while we have shown that the TSSA provided insight into the interplay between PA dimensions and other CVD risk factors and their association to CVD in specific subgroups, we did not perform complementary regression analyses. This is because the goal of our research was not to compare results yielded by the two statistical models.

Finally, we used register data to obtain information on CVD events. The unique Danish Civil Registration Number assigned to all Danish citizens allowed for accurate ascertainment of CVD events with no loss to follow-up. However, we also acknowledge the relative short follow-up period as one of the limitations of this study. Furthermore, during follow-up, 57 study participants died from causes other than CVD. However, because this number constitutes < 5% of the population, the effect of competing risk from non-CVD death was considered insignificant in this study.

The effect of PA in prevention of CVD events remains to be fully determined. As the number of ageing individuals and people with pre-diabetes and type 2 diabetes is predicted to increase, the prevention of CVD becomes even more urgent. Taken together, our prospective findings suggest that among various PA dimension, only PAEE is associated with the development of CVD. The fact that we found an association only in the subgroups at lowest risk of CVD may possibly indicate that in order to effectively reduce cardiovascular risk and events over time, any type of PA that increases PAEE should be implemented before a high risk state for CVD develops. Understanding the heterogeneity of the role of PA is important to guide personalized PA recommendations and translation of PA prevention studies to clinical and public health settings. Future prospective and clinical trials are needed to unravel and firmly establish the presumed CVD protective role of different PA dimensions, especially at any course and level of type 2 diabetes risk.

## Additional files

**Additional file 1: Table S1.** International Classification of Diseases (ICD) codes for cardiovascular disease.

**Additional file 2: Figure S1.** Tree-structured survival analysis for first cardiovascular disease event in the ADDITION PRO cohort study including CVD protective medications as risk factors.

## Abbreviations

BMI: body mass index
CI: confidence interval
CVD: cardiovascular disease
HbA1C: glycated hemoglobin A_1c_
IQR: inter-quartile range
IR: incidence rate
MET: metabolic equivalent of task
MVPA: moderate to vigorous physical activity
PA: physical activity
PAEE: physical activity energy expenditure
SD: standard deviation
TSSA: tree-structured survival analysis
