# Supplementary material for: The role of physical activity in the development of first cardiovascular disease event: a tree-structured survival analysis of the Danish ADDITION-PRO cohort
Source: Cardiovasc Diabetol. 2018 Sep 12;17:126. doi: 10.1186/s12933-018-0769-x (PMC6134699; doi:10.1186/s12933-018-0769-x)
Supplement: Supplementary file 1 — Additional file 1: Table S1. International Classification of Diseases (ICD) codes for cardiovascular disease. [file 12933_2018_769_MOESM1_ESM.docx]

**Table S1. International Classification of Diseases (ICD) codes for cardiovascular disease**

|  | **ICD 10** | **ICD 8** | **SKS codes (Danish procedure codes)** |
| --- | --- | --- | --- |
| Ischemic heart disease | DI 20-25 | 41[0-3] | KFN[A-H] covers any cardiac operation due to ischemic heart disease  **Exeptions: KFNG20 and KFNG22: Removal of foreign body in coronary artery* |
| Ischemic stroke | DI 63-66  DI 69.3-4 | 43[2-7] |  |
| Heart failure | DI 50 |  |  |
| Atrial fibrillation/flutter | DI 48 | 42793-4 |  |
| Atherosclerotic disease | DI 70  DI 73.9A+C  DI 74 | 440[0-3]  44099 | KNGQ09, 19, 29, 39, 49, 99  KNHQ00, 02, 03, 05, 07, 1, 11, 14, 17, 2, 20-25, 27, 99  Covers amputations and related operations at upper and lower leg, knee, ankle and foot level |
