# Supplementary material for: The role of physical activity in the development of first cardiovascular disease event: a tree-structured survival analysis of the Danish ADDITION-PRO cohort
Source: Cardiovasc Diabetol. 2018 Sep 12;17:126. doi: 10.1186/s12933-018-0769-x (PMC6134699; doi:10.1186/s12933-018-0769-x)
Supplement: Supplementary file 2 — Additional file 2: Figure S1. Tree-structured survival analysis for first cardiovascular disease event in the ADDITION PRO cohort study including CVD protective medications as risk factors. [file 12933_2018_769_MOESM2_ESM.docx]

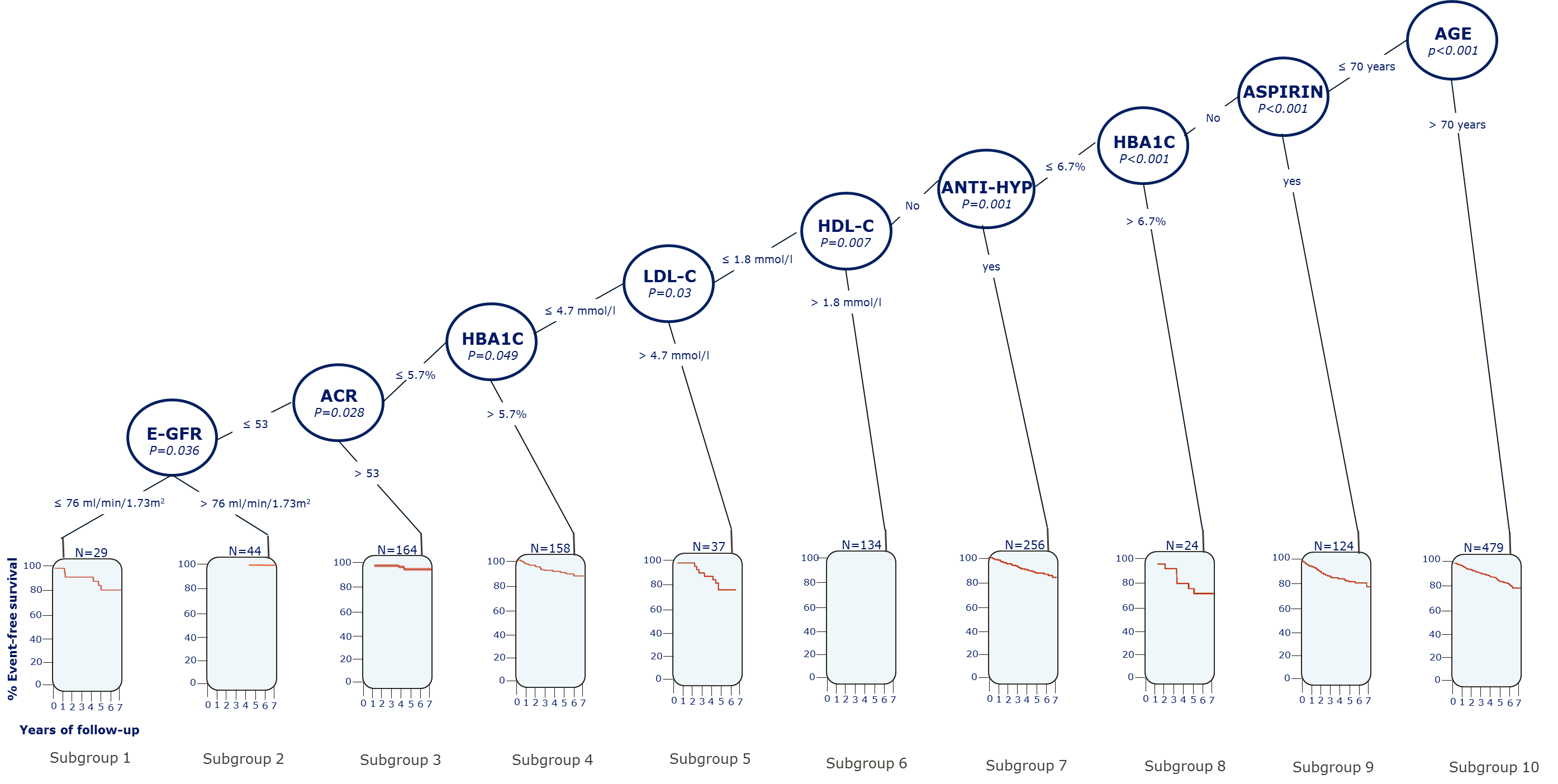


**Figure S1. Tree-structured survival analysis for first cardiovascular disease event in the ADDITION PRO cohort study including CVD protective medications as risk factors.**

*AGE: age at baseline; ASPIRIN: use of aspirin; HBA1c: glycated hemoglobin A1c; ANTI-HYP: use of any anti-hypertensive drugs; HDL-C: HDL-cholesterol; LDL: LDL-cholesterol; ACR: albumin creatinine ratio; E-GFR: estimated glomerular filtration rate*
